# Conjunctival Sparing Ptosis Correction by White-Line Advancement Technique

**DOI:** 10.1155/2020/9021848

**Published:** 2020-07-15

**Authors:** Fatima A. Habroosh, Habibullah Eatamadi

**Affiliations:** Oculoplastic Unit, Sheikh Khalifa Medical City, Abu Dhabi, UAE

## Abstract

**Purpose:**

To describe a modified technique of white line advancement posterior ptosis surgery and to report the success rate of the procedure.

**Methods:**

A retrospective case series of 60 patients who presented with ptosis with good levator function. The success rate was defined as an MRD1 of greater than or equal to 3.5 mm, symmetrical eyelid position with an intereyelid height asymmetry of ≤1 mm, and a satisfactory eyelid contour at 3 months follow-up.

**Results:**

Sixty patients (91 eyelids) met the inclusion criteria. Mild postoperative complications occurred in 11 patients that resolved without surgical intervention. Seven patients had recurrence of ptosis: four patients had early recurrence and 3 had late recurrence. The success rate was 88.33% with an average follow-up of 9 months.

**Conclusion:**

This procedure is a promising technique in cosmetic and functional ptosis correction. The advantage of this posterior approach procedure is that there is no conjunctival resection; it is suitable for young patients who do not have excess eyelid skin. The procedure is quick with a short recovery period. Additionally, it can be combined with another procedure and in different pathology.

## 1. Introduction

Involutional ptosis is the most common etiology of ptosis encountered by oculoplastic surgeons. The cause of involutional ptosis is believed to be due to dehiscence or disinsertion of the levator palpebrae superior (LPS) aponeurosis from the anterior surface of the tarsus. Reinsertion of LPS in involutional ptosis can be performed through anterior or posterior approaches. Anteriorly, an external LPS advancement surgery can be performed; this surgery has a 60–95% success rate with somewhat unpredictable postoperative height and contour [1]. Posterior approach (transconjunctival) ptosis surgery was possibly first described by Bowman in 1857 [2]. In the next century, Blaskovics may have been the first to describe this technique. In his publication, he illustrated the dissection of the levator palpebrae superioris from the surrounding tissues and shortening the muscle. In 1929, Blaskovich further modified his technique by creation of the fold and resection of the levator and part of the tarsal plate in all cases [3, 4]. This approach was further addressed by Agatston when he stated in 1942 that this technique was becoming increasingly popular in the United States [5]. By 1952, Berke had modified Blaskovich technique and included surgeries in congenital ptosis. He minimized the number of sutures to one only and did not resect the tarsal plate [6, 7]. One of the procedures that has endured test of the time is the Fasanella–Servat Procedure. Fasanella and his fellow Servat resected the conjunctiva, 2–4 mm of the superior edge of the tarsal plate, and the Muller muscle to correct mild ptosis surgery. This gained widespread popularity among ophthalmologists. They published their result in 1961 [8, 9]. Despite its popularity, many ophthalmologists and, specifically, oculoplastic surgeons were finding the resection of the tarsal plate (especially in a young patient) “destructive.” Putterman and Urist, in 1975, described their well-known technique of Müller's muscle and conjunctival resection (MMCR) in 1975. This technique depended on the positive phenylephrine test [10]. Since their original description, there have been numerous modifications to the MMCR technique. “The white-line advancement technique” has been one of these major modifications that, again, has been described by multiple authors in the recent years.

The major advantages of this technique are its undependability on the phenylephrine test, postoperative predictability, reproducibility, and long-lasting results [11, 12]. Most versions of the posterior approach ptosis repair involve varying degrees of the resection of the conjunctiva, the Muller muscle, and, in some techniques, part of the superior edge of the tarsal plate. The “white-line” advancement technique spares these tissues. The white-line term is an alternative anatomical description of levator aponeurosis. In this report, we describe our results with modifications of white-line advancement posterior ptosis surgery.

## 2. Methods

An institutional review board approval of Sheikh Khalifa Medical City was obtained, and the study adhered to the ethical principles outlined in the Declaration of Helsinki as amended in 2013. Informed consent was obtained from all the patients. A retrospective chart review of 60 patients was performed from January 2014 to September 2019, who presented with ptosis and underwent surgical correction with white-line advancement technique under local or general anesthesia. A good levator function of 12 mm or better was required for the performance of the procedure. Congenital ptosis was excluded from the study. The success rate was defined as an MRD1 of greater than or equal to 3.5 mm, maintaining a symmetrical eyelid position with an intereyelid height asymmetry of ≤1 mm and satisfactory eyelid contour at a minimum of 3 months postoperatively. Data collection was performed using Microsoft Excel and was based on the number of patients, not eyelids. All patients had consented for a full-face photography prior to and after the procedure.

### 2.1. Clinical Examination

All of the patients underwent a detailed ophthalmic evaluation that included the following: the onset and duration of ptosis, degree of ptosis documented by margin reflex distance 1 (MRD1), levator palpebrae superioris (LPS) function, tear film evaluated by tear breakup time, phenylephrine test, evaluation of extraocular movements, Bell's phenomenon, strength of the orbicularis muscle, corneal sensation, and the presence of contralateral ptosis, as well as anterior and posterior segment examination. Phenylephrine 2.5% was used in the more ptotic eye, and the response was measured and documented photographically. If this test was positive, the drooping, if any, of the contralateral upper eyelid from the baseline was also documented.

### 2.2. Surgical Technique

The procedure was usually performed under local anesthesia, but general anesthesia or sedation was an alternative option for anxious patients.

**Figure 1:**
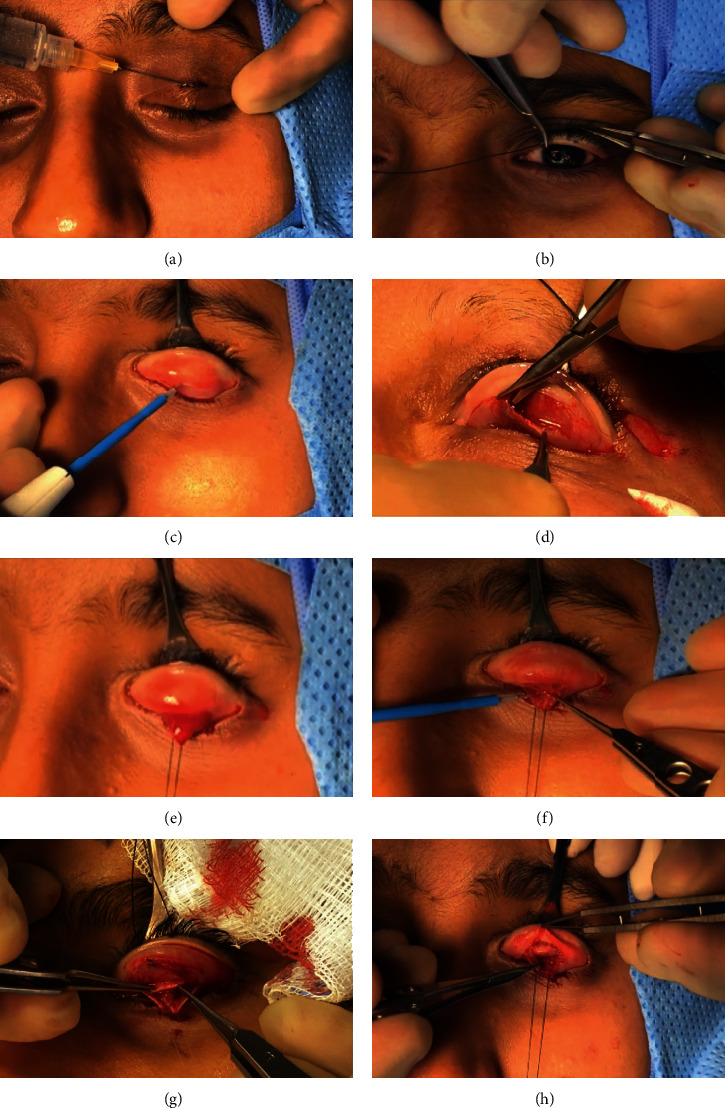
Surgical steps. (a) Infiltration of the local anesthesia intended skin crease. (b) A 4-0 silk suture was passed through the grey line of the upper lid. (c) The conjunctiva was cut 1 mm away from the upper border of the tarsal plate by monopolar. (d) Blunt dissection of the conjunctiva off the Müller muscle. (e) Conjunctival tractional suture. (f) Incision of the Müller muscle along the same line with monopolar cautery. (g) The white line is often found posteriorly. (h) Dissection of the anterior surface of the tarsal plate from the pretarsal orbicularis muscle.

**Figure 2:**
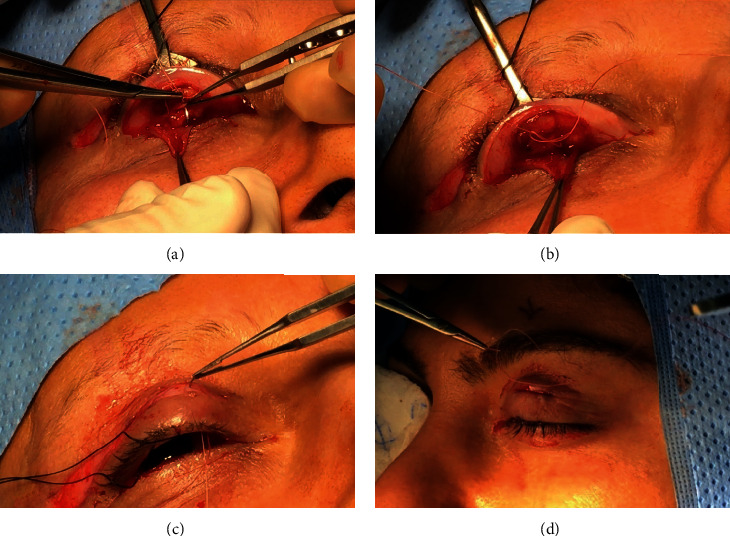
Surgical steps (continue). (a) After placing a vertical suture in the tarsal plate, the same suture was passed through the white line, incorporating no more than 1 mm of its thickness. (b) A knot (2 throws) is tied onto the anterior surface of the tarsal plate to position the levator onto it. (c) Both ends of the suture are passed under upper part of the tarsal plate and exteriorized through the initial skin incision. (d) The suture is tied over the orbicularis oculi muscle.

Prior to infiltration of the local anesthesia, the intended skin crease incision site was marked (Figure 1(a)). Additionally, the upper lid margin was marked at the level of the pupil as a guide for the placement of the suture onto the anterior surface of the tarsal plate. Approximately 2 ml of 2% lidocaine with 1 : 200000 adrenaline was infiltrated along the marked skin area and, also, subconjunctivally after eyelid eversion. Following local anesthesia, the skin crease incision was performed at the marked area (not more than 3 mm). A 4-0 silk suture was passed through the grey line of the upper lid (Figure 1(b)). The lid was everted over a DeMarre's retractor. The superior border of the upper tarsal plate was identified, and with the use monopolar cautery, the conjunctiva was cut 1 mm away from the upper border of the tarsal plate (Figure 1(c)). As the upper lid is under traction, the conjunctiva retracts back by another 1.0 mm. This provides a space for further dissection of the conjunctiva off Müller's muscle with monopolar cautery. Blunt dissection of the conjunctiva off Müller's was, then, performed with a wet cotton tipped applicator (Figure 1(d)). Next, Müller's muscle was incised along the same line with monopolar cautery and separated from the underlying levator aponeurosis (Figure 1(f)). As the dissection of Müller's muscle is continued, the “white line” becomes visible in full length. The white line is often found posteriorly (Figure 1(g)), as expected, based on the severity of the ptosis. In severe ptosis, a gentle further posterior dissection is needed to reveal the white line. The white line is grasped with 0.3 mm tipped forceps and minimally dissected off the surrounding tissue for the placement of the sutures. By using a blunt Wescott scissors, the anterior surface of the tarsal plate is, then, dissected from the pretarsal orbicularis muscle 3 mm from the upper lid margin (Figure 1(h)). A double-armed 5-0 vicryl suture is passed through the anterior surface of the tarsal plate (half-thickness), approximately 2 mm posterior to the upper tarsal margin (Figure 2(a)). The authors prefer a vertical placement of the suture to the tarsal edge. After checking that the suture is half-thickness through the tarsal plate by direct visualization, the needle is passed through the white line, incorporating no more than 1 mm of its thickness. At this stage, a knot (2 throws) is tied onto the anterior surface of the tarsal plate to position the levator onto it (Figure 2(b)). Next, both ends of the suture are passed under upper part of the tarsal plate (but not incorporating the tarsal plate) and exteriorized through the initial skin incision (Figure 2(c)). The suture is tied over the orbicularis oculi muscle (Figure 2(d)). The height and the lid contour are checked and adjusted. Rarely, a second suture is needed lateral to the first suture. However, in the majority of cases, placement of one suture suffices to achieve excellent lid contour and height. The skin is then closed with a single 7-0 vicryl or prolene suture. An eye-pad is placed for 24 hours, and suture removal is performed at 1 week. Postoperatively, the patient is instructed to use ice packs and lubricating eye drops. Any other concomitant procedures, if needed (blepharoplasty, excision of excess fat, transposition of medial fat pad, lacrimal gland repositioning, and lower lid blepharoplasty), are performed prior to the ptosis surgery using routinely performed standard operating procedures.

## 3. Results

**Figure 3:**
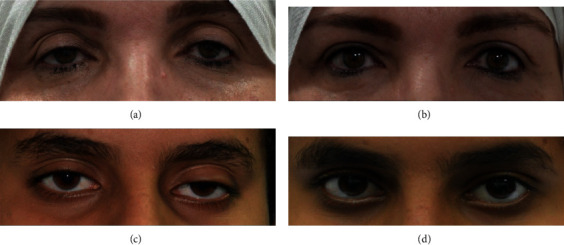
Pre- and postoperative pictures. (a) Bilateral involutional blepharoptosis. (b) One month after bilateral blepharoptosis repair by white-line advancement technique. (c) Bilateral mechanical blepharoptosis due to vernal keratoconjunctivitis. (d) One month after bilateral blepharoptosis repair by white-line advancement technique.

Ninety-one eyelids of 60 patients were enrolled in the study. There were 24 males and 36 females. The mean age was 49 years (range: 18–81 years). Ninety-four percent of patients had acquired aponeurotic blepharoptosis (79 eyelids). Other types included unilateral mechanical ptosis (due to upper eyelid and brow neurofibroma (one eyelid) and ptosis following severe vernal keratoconjunctivitis (one eyelid)) and ptosis associated with anophthalmoic socket (2 eyelids). The majority of the patients (*n* = 55) were operated under pure local anesthesia. Monitored local anesthesia (*n* = 2) and general anesthesia (*n* = 3) were the other modalities used. Ten percent of the patients had previous eyelid surgery, the majority of whom (66.66%) had a previous ptosis correction procedure. The mean preoperative MRD1 was 1.5 ± 0.5 mm. The mean levator aponeurosis function was 14 ± 1.5 mm. Mean preoperative intereyelid asymmetry in unilateral cases was 2 ± 5.0 mm. Mean postoperative MRD was 3.8 ± 0.3 mm. Mean postoperative intereyelid asymmetry was 0.3 ± 0.2 (Figure 3).

At a mean follow-up of 8 ± 2 months, successful outcomes were noted in 88.33% of the patients (Figure 3). Mild postoperative complications occurred in 11 patients, which included peaking (*n* = 3), over correction (*n* = 1), lagophthalmos (*n* = 1), under correction (*n* = 7), suture granuloma (*n* = 2), and hematoma (*n* = 3). Most resolved without the need for further surgical intervention. Three out of 7 patients who had undercorrection required further surgical procedures. Analysis for those with suboptimal results revealed one had a severe postoperative hematoma and one had wound infection. Of the repeat procedures, one patient had revision ptosis correction with posterior white-line advancement, and two had external levator advancement with successful outcomes. The other four patients were lost to follow-up. Concomitant procedures performed included upper eyelid blepharoplasty with or without fat excision/fat transfer (*n* = 19), lacrimal gland repositioning (*n* = 3), and bilateral lower lid blepharoplasty (*n* = 3). The patient with neurofibromatosis underwent initial debulking of the eyelid and brow neurofibroma. The residual ptosis was corrected with a conjunctival approach of white line advanced 6 months after the initial surgery. The result of the surgery was sustained for 2 years following the ptosis surgery.

The mean operative time was 15 ± 5 minutes per eyelid when the procedure was performed alone. Concomitant surgeries added on average 18 ± 8 min to the operating time.

Based on our experience, the down time of external ptosis correction was about 2 weeks (ranging from 7–14 days), in contrast to white-line advancement technique which was about 1 week (ranging from 3–7 days).

## 4. Discussion

Predictability of the surgical outcome in blepharoptosis is possibly one of the most important factors both to the patient and the surgeon. There are many variables when assessing the outcome of any ptosis surgery. The ideal preferred technique would be the one that is highly predictable, repeatable, easy to perform, associated with minimal complications, and has a short postoperative recovery period. The preferred techniques of ptosis surgery have evolved over time. External aponeurosis advancement has been practiced over decades and results in a wide success rate of 65–90% [13–15].

The success rate of different types of posterior approach ptosis correction has been reported to be higher, with better predictability [7–10]. In his original description, Putterman reported a success rate of 90%, defined as a symmetry of up to 1.5 mm difference between the two eyelids. However, the prerequisite for inclusion was a positive phenylephrine test [16].

Others have reported a success rate of 85%–98% [11, 17]. Malhotra et al. reported a success rate of 87% with good eyelid contour. In this series, the Muller muscle was not resected, and the decision to operate did not depend on a positive phenylephrine test [11]. Similar criteria and success rates were also reported by Lake et al. [11, 17, 18].

Our study yields a similar success rate of 88.33%. Two of our recurrences were due to postoperative bleeding and infection. All other postoperative complications needed minimal intervention (excision of the granuloma, short term gentle massaging of the upper eyelid for mild overcorrection, or peaking) or no intervention.

The current study also combines posterior approach white-line levator advancement with other surgeries and in the presence of coexisting pathologies with satisfactory outcomes. All of our patients achieved excellent postoperative results including those with neurofibromatosis, anophthalmic socket, and ptosis as a result of severe vernal keratoconjuncivitis. Though the number of such patients is low, this opens the window to increase the indication for this procedure to be performed in conditions other than simple aponeurosis dehiscence.

A response to phenylephrine testing has been argued to be a prerequisite for choosing conjunctival approach ptosis surgery. Baldwin et al. reported excellent results irrespective of this test [17]. The same conclusion was drawn by Malhotra et al. The present series also reaffirmed that white-line advancement does not need a positive phenylephrine test in patients with a levator function of 12 and more [12]. The authors believe that a healthy levator maintains its normal anatomy and function after being disinserted from its normal anatomical location. Hence, repositioning of the levator to its original anatomical position should result in regaining the normal position of the eyelid. As the Muller muscle is not excised, its function is also retained with possible secondary rearrangement of its fibers as a consequence of advancing the levator aponeurosis muscle.

Complications related to the exposed suture is minimal, mainly due to the fact that the conjunctiva is not shortened, and at the end of the surgery, the conjunctiva covers the sutures, minimizing keratopathy, and granuloma formation.

The operative time is much shorter in the current technique compared to the external approach. For the authors, the mean operating time to complete one eyelid was 15 ± 5 min, when not combined with any other procedures. This is much shorter when compared to the earlier reported durations using either external or transconjunctival approaches [19].

Time off work is another important factor that has an economic impact on the patient and on financial entities. In our study, the maximum given sick leave was 7 days including the weekend. This is significantly shorter than the time taken to recover following external approach ptosis surgery which, as per our experience, is averaged at 13 ± 1 days.

Many surgeons have developed an algorithm for the posterior approach ptosis correction, and some of these techniques rely on the positive phenylephrine test [18, 20, 21]. We postulate that as our technique involves direct incorporation of the levator aponeurosis and does not depend on the Muller muscle, such algorithms are not necessary to achieve a satisfactory postoperative result.

Our series included conditions other than aponeurotic dehiscence (mechanical ptosis and in the setting of an anophthalmic socket). Fleming et al. have reported similar results in 5 patients, but their surgery involved mullerectomy at the same time [22]. The combination of white-line advancement with upper lid blepharoplasty and lacrimal gland prolapse repositioning demonstrated excellent results in a select group of cases.

The initial skin incision serves an important function. Following a 2-3 mm skin incision, a pocket of space is created between the fibers of orbicularis, and this space is used to bury the vicryl knots, instead of externalizing the knot on the skin. The skin is subsequently closed with 7/0 or 8/0 vicryl. This modification minimizes the cases of wound infection or suture-related abscess and granuloma.

There have been multiple studies reporting the result of this procedure in North American, European, and Far East Asian population. This the first study of its kind to look into this procedure in the population from the Middle Eastern population.

The number of eyelids in this study is one of the highest among published. However, the limitation of our study is that it is a retrospective in nature, noncomparative, and the sample size of subgroups is small, not allowing meaningful subgroup comparisons.

In conclusion, the white-line advancement conjunctival approach ptosis surgery is gaining popularity with better predictability and improved outcome in terms of symmetry, eyelid lift, more physiological contour, and lesser operative and recovery time.

## Data Availability

We cannot guarantee full data access due to the ethical consideration of our institute.

## Supplementary Materials

Supplementary Materials Video describing the surgical steps.
